# The Study of Metal Sulfide as Efficient Counter Electrodes on the Performances of CdS/CdSe/ZnS-co-sensitized Hierarchical TiO_2_ Sphere Quantum Dot Solar Cells

**DOI:** 10.1186/s11671-017-1926-y

**Published:** 2017-03-07

**Authors:** Nattha Buatong, I-Ming Tang, Weeraphat Pon-On

**Affiliations:** 1 Department of Physics, Faculty of Science, Kasetsart University, Bangkok, 10900 Thailand; 2 Department of Material Science, Faculty of Science, Kasetsart University, Bangkok, 10900 Thailand

**Keywords:** Sensitized solar cell, Quantum dots, Hierarchical titanium dioxide, Counter electrodes, Copper zinc tin sulfur (selenium) (CZT(S,Se))

## Abstract

The effects of using different counter electrode metal sulfides on the performances of solar cells made with CdS/CdSe/ZnS quantum dots co-sensitized onto hierarchical TiO_2_ spheres (HTSs) used as photo-electrode are reported. The HTS in the QDSSCs is composed of an assembly of numerous TiO_2_ spheres made by the solvolthermal method. The photoelectrical performance of HTS/CdS/CdSe/ZnS coupled to CuS or to Cu_2_ZnSn(S_1 − *x*_Se_*x*_)_4_ with *x* = 0, 0.5, or 1.0 counter electrodes (CEs) were compared to those coupled to Pt CE. The HTS/CdS/CdSe/ZnS coupled to the CuS CE showed the highest power conversion efficiency *η* (of 3.46%). The efficiencies *η* of 1.88, 2.64, and 2.06% were obtained for CZTS (*x* = 0), CZTS_0.5_Se_0.5_ (*x* = 0.5), and CZTSe (*x* = 1), respectively. These are significantly higher than those using a standard Pt CE (*η* = 0.37%). These higher efficiencies are the results of the higher electrocatalytic activities when the metal sulfide CEs are used.

## Background

Semiconductor quantum dots (QDs) have received a great attention as a new photovoltaic material due to their extraordinary optical and electrical properties (e.g., a tunable band gap and high molar extinction coefficient, respectively) as well as having high theoretical photovoltaic conversion efficiencies (up to 44%) [1–6]. A typical configuration of quantum dot-sensitized solar cells (QDSSCs) used as a photovoltaic device consists of QDs absorbed on the wide band gap semiconductor TiO_2_ (or ZnO) assembled on a platinum (Pt) counter electrode (CE) [4–6]. The use of CdS, CdSe, CdTe, and PbS QDs sensitized for light harvesting in QDSSCs has been widely studied because of their high potential for photo absorption in the visible light region and their easy preparation [4–6]. Under illumination, photo excitation of the semiconducting QDs is followed by electron injection into the conduction band of a wide band gap semiconductor.

In the last few years, the conversion efficiencies for organic solar cells have been improved so that they can be now higher than those of semiconductor solar cells. The major obstruction to improving the conversion efficiency of QDSSCs is the difficulty in assembling the semiconducting QDs onto the TiO_2_ matrices, the charge recombination which occurs at the interface between the photoanode and the electrolyte and to the poor electrical catalytic activity between the polysulfide electrolyte and the counter electrode. To overcome these obstacles, one needs to optimize the charge injection from the QDs to the TiO_2_ photoanodes and the increasing of the redox coupling at the counter electrode [7–18]. The chief goals are to increase the carrier generation and to inhibit the charge recombination. These can be achieved by having more ordered structures such as those which accompany the use of one-dimensional nanostructures and the use of hierarchical TiO_2_ nanorods [7–11]. The use of ordered structures leads to an increase in the electron transport and to a reduction of the recombination. In addition, the use of hierarchical spheres would increase the surface area thus maximizing the adsorption by the sensitizers and enhance the light scattering. The consequences of these two processes would be an increase in the light harvesting capability of the photo anodes. The next obstacle in QDSSCs is the poor electrical catalytic activity between the electrolytes $\left({\mathrm{S}}^{2-}/{\mathrm{S}}_x^{2-}\right)$ and the commercial Pt counter electrode. This issue has been the focus of much research in recent years [12–22]. It has been determined that Pt counter electrodes should not be used when polysulfide electrolytes are used since the sulfur compounds are strongly adsorb to the Pt surface, thus leading to decreasing catalytic activity. For this reason, metal sulfides such as Cu_2_S, CuS, CoS, PbS, NiS, CoS/NiS, multi-elemental chalcogenide (Cu_2_ZnSn(S_1 **−** *x*_Se_*x*_)_4_), earth-abundant Cu_2_SnSe_3_ and other carbon-based materials have been used as novel counter electrodes. This would lead to higher power conversion efficiencies of the QDSSCs (*η* around 3%) [12–22].

**Fig. 1:**
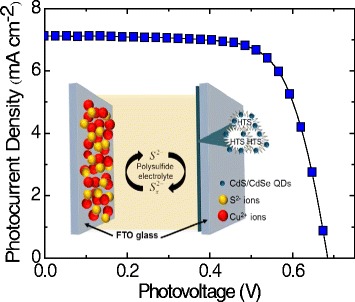
Schematic diagram of the photovoltaic performance of quantum dot (QD)-sensitized solar cell consisting of CdS/CdSe/ZnS QDs loaded onto the surface of the hierarchical TiO_2_ spheres assembling CuS and CZT(S_1 **−** *x*_Se_*x*_)_4_ counter electrodes

Based on the potentially larger surface area of the three-dimensional hierarchically structured sphere of TiO_2_ and remarkable boost of the catalytic activity of metal sulfide materials, we have made QDSSCs in which the HTS/CdS/CdSe/ZnS photoanodes are coupled to counter electrodes made with CuS or Cu_2_ZnSn(Sn_1 **−** *x*_Se_*x*_)_4_ (*x* = 0, 0.5, and 1) as shown in Fig. 1. A QDSSC using Pt was also constructed for reference purposes. The QDSSCs made with last three counter electrodes are denoted as CZTS (*x =* 0), CZT(S_0.5_Se_0.5_) (*x* = 0.5) and CZTSe (*x* = 1.0), respectively. The structure, morphologies, and optical properties of the QDSSCs having the novel counter electrodes were characterized using X-ray diffraction (XRD), scanning electron microscopy (SEM), transmission electron microscopy (TEM), and UV–vis spectroscopy. To determine the photovoltaic performance of the QDSSCs containing the HTS/CdS/CdSe/ZnS photoanodes in aqueous polysulfide electrolytes $\left({\mathrm{S}}^{2-}/{\mathrm{S}}_x^{2-}\right)$, the photocurrent-voltages (*J–V*) curves of these cells were obtained. These curves were then analyzed to determine the performance efficiencies. In this study, an efficiency of *η* of 3.46% has been achieved for the combining CdS/CdSe/ZnS QD-loaded HTS assembling CuS counter electrode (around 9.35 folds compared with Pt CE).

## Methods

### Synthesis of Hierarchical TiO_2_ Sphere and Preparation of the Electrodes

The synthesis of the hierarchical TiO_2_ spheres (HTSs) began with the synthesis of the TiO_2_ nanoparticles using the solvothermal method given in a previous paper [23]. In a typical synthesis, 0.45 M titanium butoxide (TBT), 0.90 M hydrochloric acid (HCl), nitric acid (HNO_3_), and acetic acid (HAc) (CH_3_COOH) were added to 25 mL of *n*-hexane and stirred at room temperature for 30 min. The mixture was then transferred to a Teflon-lined stainless steel autoclave and heated to 150 °C for 5 h. The autoclave was then cooled down to room temperature naturally. After completion of the solvothermal reaction, the white precipitate was separated by centrifugation, washed with absolute ethanol two times, and dried in ambient air at 80 °C for 12 h. For preparation of the HTS electrode, the HTS paste was screen-printed onto a FTO glass plate using a doctor-blading method. The thickness of TiO_2_ films was controlled by using a layer of adhesive tape (3M Scotch brand) as a frame and spacer. All films were heat treated at 450 °C for 1 h and cooled to room temperature. The substrates were dried in desiccators before commencing with QD attachment.

### Preparation of CdS/CdSe Co-sensitized HTS Electrodes

The method of successive ion layer absorption and reaction (SILAR) was used to assemble the CdS/CdSe quantum dots on the HTS electrodes. To grow the CdS, the HTS electrodes are first immersed in a solution containing 0.04 M Cd(NO_3_)_2_ in ethanol for 5 min. In this process, the Cd^2+^ ions were absorbed onto the HTS electrodes. Then, the electrodes are rinsed in ethanol and dried with N_2_. Then the electrodes were dipped into a solution containing 0.04 M Na_2_S in a mixture of water and methanol (1:1, *v*/*v*) for 5 min. The S^2−^ ion will react with the pre-adsorbed Cd^2+^ leading to the formation of CdS. The electrodes were then rinsed with methanol and dried again in N_2_. This procedure was referred to as a one SILAR cycle. This step was repeated three times in order to achieve a suitable CdS loading on the HTS electrodes. In a sequent step, the CdSe were deposited on the CdS-coated HTS electrodes. The CdS-coated HTS electrodes were immersed in an aqueous solution of 0.1 M Cd(NO_3_)_2_ in ethanol for 5 min, rinsed with ethanol, and dried with N_2_. The electrodes were then immersed in 0.1 M Na_2_SeSO_3_ solution containing water and methanol (1:1, *v*/*v*) for 5 min. The electrodes were then rinsed in methanol and dried. This was repeated three times to obtain the CdSe on the Cd-coated HTS electrodes. Finally, a ZnS passification layer was deposited by two immersions in a 0.1 M Zn(N O_3_)_2_ solution and in a 0.1 M Na_2_S aqueous solution. The two solutions served as the sources of the Zn^2+^ and S^2−^, respectively. The dipping for 1 min in each solution lasted 1 min each time.

### Synthesis and Preparation of the Counter Electrodes of CuS and Cu_2_ZnSn(S_1 **−** *x*_Se_*x*_)_4_ (*x* = 0, 0.5, and 1)

#### CuS Synthesis and Counter Electrode Fabrication

The CuS counter electrodes were prepared using the method given in the literature [24]. For the preparation of the CuS electrodes, a doctor-blade method was used and was described as follows: 0.5 M Cu(NO_3_)_2_ in a methanol solution (100 μL) was first dropped onto a FTO glass plate. Then 1 M Na_2_S in a mixture of water and methanol (3:7, *v*/*v*) aqueous solution (100 μL) was dropped on the layer of Cu(NO_3_)_2_-decorated FTO glass plate. This resulted in an immediate color change form blue to brown and then finally to black, indicating the formation of the CuS. The remainder of the ions was removed by rinsing with ethanol. The plate was then dried in ambient air. This procedure is referred to as being one cycle. Four cycles were used to deposit a suitable amount of CuS. The films were then heat treated at 120 °C for 30 min under ambient air condition, followed by cooling to room temperature.

#### Cu_2_ZnSnS_4_ Synthesis and Counter Electrode Fabrication

The Cu_2_ZnSnS_4_ (CZTS) nanocrystals were prepared as follows: 0.1 mmol of copper (II) acetate, 0.05 mmol of zinc acetate, and 0.05 mmol tin (IV) acetate were mixed together in 10 ml of oleylamine (OLA). The mixture was then heated under vacuum to 120 °C to remove any traces of water and to completely dissolve the precursors (OLA metal precursors). After degassing at 120 °C for 30 min, the temperature was raised to 230 °C. A solution of OLA-S was prepared through the sonication of 0.05 mmol 1-dodecanethiol (1-DDT) in 1.25 mL OLA. The OLA-S solution was then injected into the OLA metal precursor solution. The mixture was mixed continuous for a further 30 min. The mixture was naturally cooled down to room temperature. The CZTS nanocrytals were washed with methanol two to three times and centrifuged at a speed of 6000 rpm for 5 min to yield a centrifuged product. Finally, the CZTS nanocrytals were deposited onto a FTO glass substrate using a commercially available 3M scotch tape to define the active area of CZTS nanocrystal thin films. The films were then heated at 400 °C for 30 min.

#### Cu_2_ZnSn(S_1 **−** *x*_Se_*x*_)_4_ (*x* = 0.5 and 1) Synthesis and Counter Electrode Fabrication

Cu_2_ZnSn(S_1 **−** *x*_Se_*x*_)_4_ nanocrystals with *x* = 0.5 and 1 were prepared using the method given in ref. [20]. In a typical synthesis, Se powders of different concentrations were added to 3 mL of OLA and heated at 240 °C for 30 min under N_2_ flow to form the OLA-Se solution. To the OLA-S solution, 1-dodecanethiol (1-DDT) of different concentrations was dissolved in 1.25 mL OLA using sonication to mix the solution. To prepare the OLA metal precursor solution; copper (II) acetate (0.1 mmol), zinc acetate (0.05 mmol), and tin (IV) acetate (0.05 mmol) were mixed in 19 mL of the OLA and heated under vacuum to 120 °C for 30 min. The mixture was then cooled down to room temperature. The OLA-S solution was then injected into the OLA-Se solution at room temperature to obtain the OLA-metal precursor solution. The CZT(S,Se) nanocrytals were allowed to grow for 30 min under N_2_ flow. Then the growth of the CZTSSe nanocrytals was terminated by removing the heating mantle and allowing the solution to cool down to room temperature. The synthesis powders were washed two or three times with methanol and centrifuged at 6000 rpm for 5 min. For the fabrication of the electrodes, the CZT(S,Se) powders were deposited on FTO glass substrates using a commercially available 3M scotch tape to define the active area of the counter electrode. Finally, the films were heated at 400 °C for 30 min.

### Solar Cell Assembly

The cells were assembled by placing the different photo electrodes over the different counter electrodes. These electrodes were separated by a 60-μm-thick thermoplastic biphenyl frame (Surlyn). The polysulfide electrolyte was introduced through a hole which was then sealed by a Ti foil. The polysulfide electrolyte was prepared by mixing 0.6 M Na_2_S, 0.2 M S, and 0.2 M KCl in a mixture of water and methanol (3:7, *v*/*v*). The active cell area is 0.25 cm^2^.

### Characterization

The crystalline phase was characterized by X-ray diffraction (XRD; Bruker D8 Advance). Diffraction patterns were recorded in the range of 20–80° at a scanning speed of 0.02° s^−1^. The morphology and structure of the samples were characterized by a scanning electron microscopy (SEM; JEOL JSM-6301F) and a transmission electron microscopy (TEM; JEOL JSM-2010). The absorption spectra were studied with a UV–vis spectrophotometer (Perkin Elmer Lambda 900). The specific surface areas of hierarchical TiO_2_ sphere (HTS) substrates were determined by the nitrogen adsorption-desorption isotherm measurement (Autosorb-cl analyzer (Quantachrome Instruments)). The total pore volume was determined at *(P*/*P*0) 0.99. The photocurrent-voltages (*J–V*) curves of the cells were measured under AM 1.5G simulated sunlight produced by a 150-W Class A Solar Simulator (Model 92250A, Oriel) at an illumination intensity of 100 mW cm^−2^. The photovoltaic performance, filling factor (FF) and power conversion efficiency (*η*) of the different QDSSCs are calculated according to the following equations [5]:

1 $$ \mathrm{F}\mathrm{F}=\frac{P_{\max }}{J_{\mathrm{sc}}\times {V}_{\mathrm{oc}}}=\frac{J_{\max}\times {V}_{\max }}{J_{\mathrm{sc}}\times {V}_{\mathrm{oc}}} $$

2 $$ \eta \left(\%\right)=\frac{P_{\max }}{P_{\mathrm{in}}}\times 100\%=\frac{J_{\mathrm{sc}}\times {V}_{\mathrm{oc}}\times \mathrm{FF}}{P_{\mathrm{in}}}\times 100\% $$

where *P*_max_ is the maximum power output, *P*_in_ is the incident light power, *J*_sc_ is the short circuit current density (mA cm^−2^), *V*_oc_ is the open circuit voltage (*V*), and *J*_max_ (mA cm^−2^) and *V*_max_ (*V*) are the current density and the voltage at the point of maximum power output in the *J*–*V* curves, respectively.

The electrochemical impedance spectroscopy (EIS) was characterized in dark conditions at a forward bias, where the applied voltage was −0.5 V. A 10-mV AC sinusoidal signal was employed with a constant bias over *z* frequency range between 0.03 Hz and 1 MHz.

## Results and Discussion

### Structure, Morphology, and Optical Characterization of the Photoanode

**Fig. 2:**
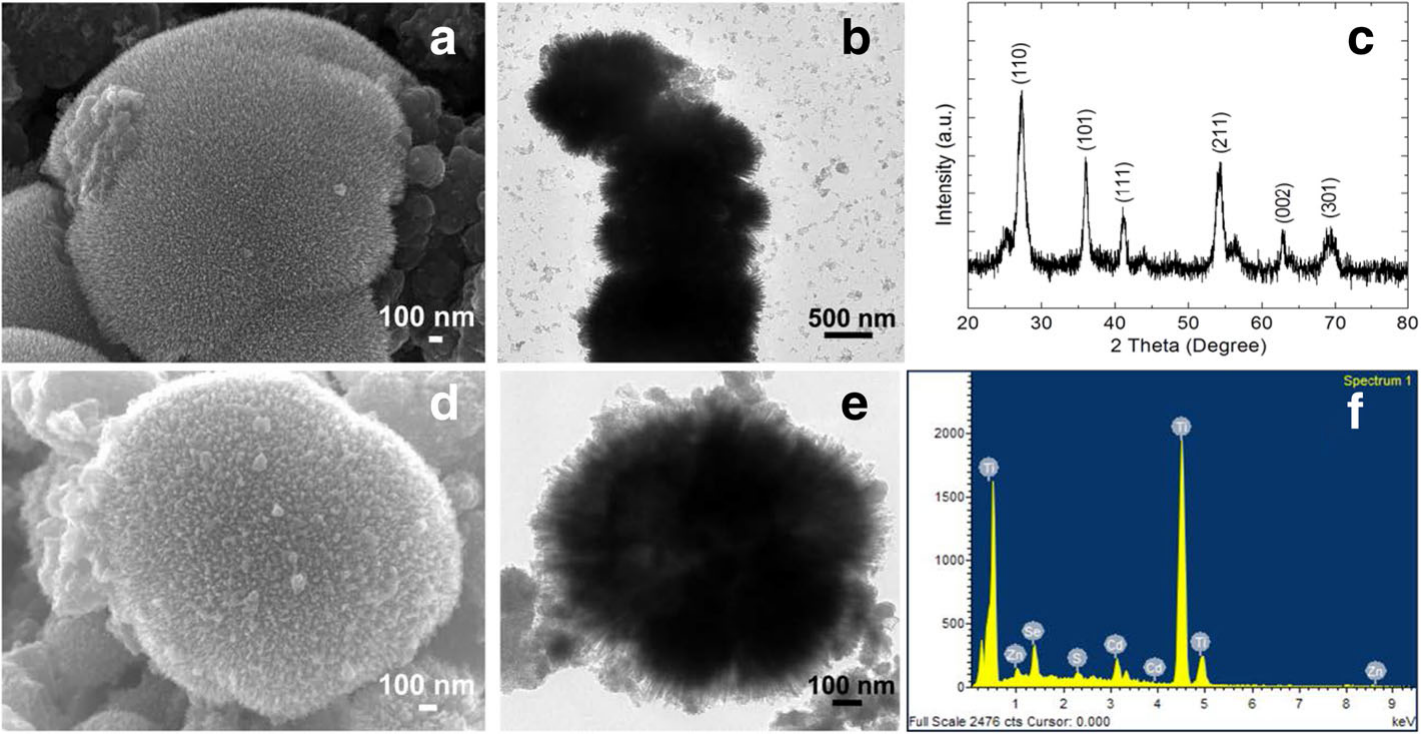
SEM (**a**) and TEM (**b**) images of hierarchical TiO_2_ spheres (HTSs). **c** XRD pattern of HTS and **d, e** SEM and TEM images of CdS/CdSe/ZnS QDs sensitized on HTS and EDS analysis of the deposited ion species of the QDs on HTS substrates (**f**)

The SEM images of the hierarchical TiO_2_ spheres (HTSs) synthesized via a solvothermal process using an acid medium are shown in Fig. 2a. The obtained powders have three-dimensional (3D) spherical structures composed of numerous crystalline nanorods radiating from the center to form a hierarchical TiO_2_ spherical structure. The TEM images (Fig. 2b) clearly show that the TiO_2_ spheres are composed of the smaller TiO_2_ nanorods which act as building blocks to form the hierarchical spherical shapes of a few microns in size. This unique architecture has several advantages such as a large surface area, providing for superior QD adsorption and light-scattering ability which leads to a significant improvement in the power conversion efficiency [10, 11]. The growth of nanorods into spherical shaped surfaces can be understood in terms of shape controlled chemistry. It is believed that the formation of the morphology and crystal structure depends on the presence of diverse ions (Cl^−^, NO^−^_3_, and CH_3_COOH^−^) during the synthesis [23]. Figure 2c shows the XRD patterns of the hierarchical TiO_2_ spheres. All the peaks in the pattern of the HTS are found to be those of the tetragonal phase of rutile TiO_2_ (JCPDS no. 70–7347). The intensity of the (110) peak indicates that the HTS are well crystallized and grew in the [001] direction with the growth parallel to the *c*-axis. After the CdS/CdSe/ZnS sensitization, the HTS surface can clearly be seen to have a roughen texture (Fig. 2d). Figure 2e shows the TEM image of a CdS/CdSe/ZnS-sensitized HTS. Most of the QDs seen in the image appear to be individual QD and not agglomerations of them. Only partially uncovered surfaces were seen. This implied that the coverage of the surface would be superior if HTS are used. The composition of CdS/CdSe/ZnS QDs deposited onto the HTS was determined by dispersive X-ray spectroscopy (EDS). The EDS analysis (shown in Fig. 2f) exhibits the peaks of Ti, Cd, S, Se, and Zn elements. This can be viewed as being evidence that the SILAR process of assembling results in the successful deposition of the QDs on the surface of the HTS.

**Fig. 3:**
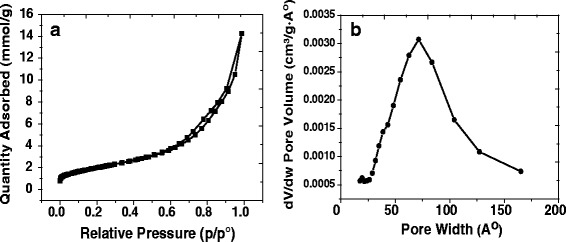
**a** N_2_ sorption isotherms and **b** pore size distribution plots of the hierarchical TiO_2_ spheres (HTSs)

The Brunauer-Emmett-Teller (BET) method is the standard method to determine the surface characteristics of porous materials. The BET method was used to determine the pore volume and area distribution of the hierarchical TiO_2_ spheres (HTSs). The hysteresis loops in the N_2_ adsorption-desorption isotherm curves are associated with the filling and emptying of the mesopores. The presence of the hysteresis loops in isotherms for the HTSs in this study (Fig. 3a) clearly shows that the formation of well-defined mesopores in the surface of the HTSs has occurred. The surface area of the HTSs used in our solar cells calculated from the isotherms is 164.23 m^2^ g^−1^. The distribution of the adsorption pore diameter of the HTS sample is shown in Fig. 3b. The distribution curve leads to the pores having an average diameter of 8.86 nm (88.62 Å). This is in good agreement with the TEM images (Fig. 2b). These values make it likely that the small QD particles will spread into the hierarchical TiO_2_ spheres interior through the mesoporous shells.

**Fig. 4:**
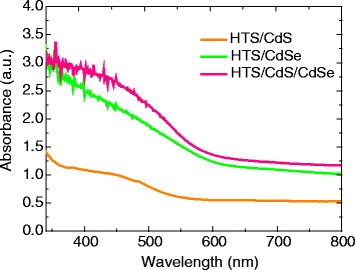
UV–vis absorption spectra of the photoanodes

The optical absorption of the QD-loaded HTS photoanodes was investigated by UV-visible absorption. The absorption spectra (Fig. 4) of the CdS, CdSe, and CdS/CdSe-co-sensitized HTS photoanodes could be clearly observed to shift in visible region. The absorption edge of the HTS/CdS electrode is located at around 500 nm, while the edge in the HTS/CdSe electrode moves to the long wavelength (around 570 nm). The explanation is that the band gap of CdSe is narrower than that of CdS. In the case of the CdS/CdSe-co-sensitized HTS electrode, the absorption range is close to that of HTS/CdSe electrode but the absorbance in the whole UV-visible region is higher than those of HTS/CdS and HTS/CdSe electrodes. The higher absorbance of the HTS/CdS/CdSe-co-sensitized electrode, compared with the HTS/CdSe, indicates that more CdSe can be loaded on the HTS/CdS/CdSe-co-sensitized electrode than on the HTS/CdSe electrode.

### Morphology of Counter Electrode

**Fig. 5:**
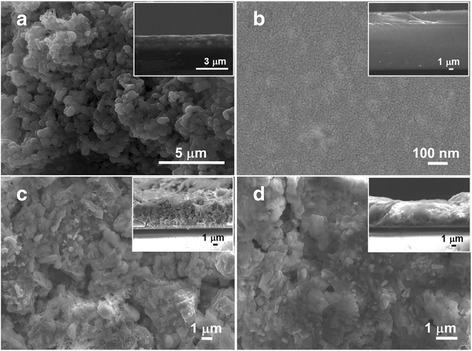
SEM images of top view and cross section of **a** CuS and Cu_2_ZnSn(S_1 **−** *x*_Se_*x*_)_4_ with *x =* 0 (**b**), 0.5 (**c**), or 1.0 (**d**) counter electrodes

From our knowledge, Pt counter electrode should not be used when polysulfide electrolyte is used since the sulfur compounds are strongly adsorb to the Pt surface, thus leading to decreasing catalytic activity and its stability tends to be reduced in QDSSCs. The SEM images of four main types of prepared counter electrodes are shown in Fig. 5. Figure 5a shows the surface morphology of CuS CE and cross-sectional image (inset) on FTO glass indicating agglomeration of small particles to form nanopore structure which renders it highly catalytically active in the CE. In case of CZT(S_1 **−** *x*_Se_*x*_)_4_ CEs, the surface morphology of the *x* = 0 (CZTS) substrate (Fig. 5b) appears mainly as a fine particle and smooth surface. However, at *x* = 0.5 (CZTS_0.5_Se_0.5_), the particles on the FTO substrate begin to form larger particles and having irregular shape with porous structure (Fig. 5c). Figure 5d shows the SEM image of CZTSe (*x* = 1) morphology onto a FTO glass substrate. The particles are promoting bigger (crystal growth) and closely packed nanoparticles. In this way, lead to narrow pore structure of the film.

**Fig. 6:**
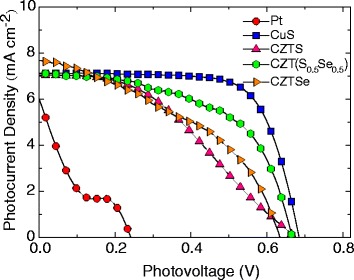
The photocurrent-voltage (*J–V*) curves of the assembled QDSSCs having the HTS photoanodes on the Pt, CuS, and Cu_2_ZnSn(S_1 **−** *x*_Se_*x*_)_4_ counter electrodes

The changes in photovoltaic performance of CdS/CdSe/ZnS-co-sensitized HTS QDSSCs having CuS, Cu_2_ZnSn(S_1 **−** *x*_Se_*x*_)_4_ (*x* = 0, 0.5, and 1) CEs, and standard Pt CEs are seen in the photocurrent-voltage (*J*–*V*) characteristics curves shown in Fig. 6. The corresponding *J–V* plot of the different CEs is given mainly attenuated by the total series resistance of the cell, electron transport resistance through the photoanode, ion transport resistance, and charge transfer resistance at the counter electrode [20, 21]. The performance parameters such as the short circuit current density (*J*_sc_), open circuit voltage (*V*_oc_), fill factor (FF), and power conversion efficiency (*η*) are listed in Table 1. These values indicate that the CdS/CdSe/ZnS-co-sensitized HTS photoanode employing CuS CE has better performances than that of QDSSCs having Pt as their CEs (higher values of *J*_sc_, *V*_oc_, FF, and *η*).

**Table 1:** Summary of the measured photovoltaic parameters of the assembled HTS/CdS/CdSe/ZnS having Pt, CuS, and CZT(S,Se) counter electrodes

Cell structures	*J*_sc_ (mA cm^−2^)	*V*_oc_ (mV)	FF	Eff (%)
HTS/CdS/CdSe/Pt	6.05	242	0.20	0.37
HTS/CdS/CdSe/CuS	7.12	683	0.71	3.46
HTS/CdS/CdSe/CZTS	7.04	674	0.40	1.88
HTS/CdS/CdSe/CZT(S_0.5_Se_0.5_)_4_	7.11	665	0.55	2.64
HTS/CdS/CdSe/CZTSe	7.68	638	0.42	2.06

The HTS/CdS/CdSe/ZnS QDSSCs having a CuS counter electrode exhibit the highest power conversion efficiency (*η)* of 3.46% owing to its short circuit current (*J*_sc_) of 7.12 mA cm^−2^, 0.683 V, and FF of 0.71. These values are higher than those of others. The QDSSCs employing CZT(S_0.5_Se_0.5_) as the counter electrode have an efficiency of 2.64% with the values of *J*_sc_, *V*_oc_, and FF being 7.11 mA cm^−2^, 0.674 V, and 0.55, respectively. The efficiency of the QDSSCs having CZTS and CZTSe as their CEs are 1.88 and 2.06%, respectively. From the data on QDSSCs having Cu_2_ZnSn(S_1 **−** *x*_Se_*x*_)_4_ as their CEs, one finds that photocurrent-voltage characteristics are superior as less Se replaces the S. The reason for this is that Se replacement lowers the electrocatalytic activity of the polysulfide solution, thereby lowering the FF and *η* of QDSSCs. To affirm this claim, Cao et al. [20] demonstrated that the reduction of polysulfide $\left({\mathrm{S}}_x^{2-}\right)$ to sulfide (S^2 −^) depended on the ratios of S/Se and that Cu_2_ZnSn(S_0.5_Se_0.5_)_4_(S/Se = 1:1) was better at this than CZTS or CZTSe and yields power conversion efficiency of 3.01%. For comparison, the HTS/CdS/CdSe/ZnS QDSSCs having Pt as their CEs have significantly lower photovoltaic performance (*η* = 0.37%, *J*_sc_ = 6.05 mA cm^−2^, *V*_oc_ = 0.242 V, and FF = 0.20). Part of the reason for the poor performance is that Pt is not a good catalyst in polysulfide electrolytes. This leads a much higher over potentials for electrolyte regeneration, resulting in low fill factor and conversion efficiency.

**Fig. 7:**
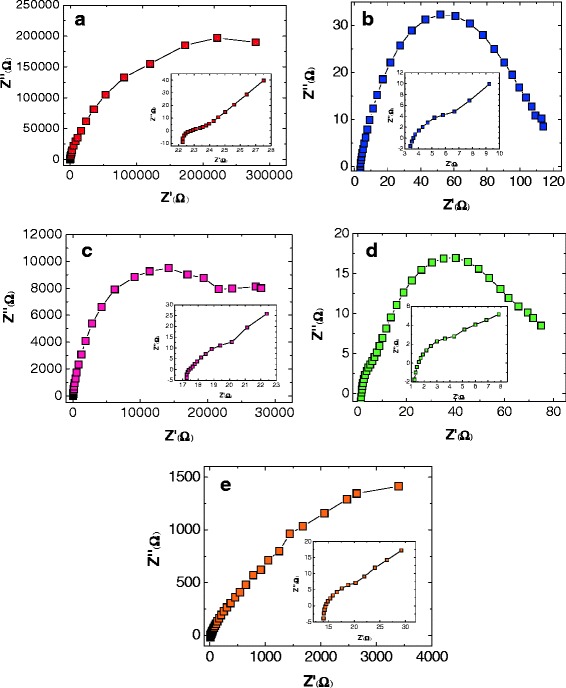
Impedance spectra of the assembled QDSSCs having the HTS photoanodes on the Pt (**a**), CuS (**b**), and Cu_2_ZnSn(S_1 **−** *x*_Se_*x*_)_4_ with *x =* 0 (**c**), 0.5 (**d**), or 1.0 (**e**) counter electrodes

From the result shown in Table 1, we see that the *V*_oc_ of Pt CE is lower than those of the others. This is due to the fact that Pt CE has a higher electron recombination rate. With the CuS CE, the value of the *V*_oc_ is 0.683 V, which is slightly larger than that with the CZT(S,Se) CEs. This is mainly due to the better conductivity and the more porous structure of CuS CE. These features lead to highly catalytic activity in the CuS CE. As a result, the electron recombination is decrease. This decrease is related to the EIS data (shown in Fig. 7). This result implies that the CuS is superior to Pt and CZT(S,Se) for use as the counter electrode when the electrolyte contains polysulfide.

In our study, we find that the performance of CuS and Cu_2_ZnSn(S_1 **−** *x*_Se_*x*_)_4_ (*x =* 0.5) as their CEs in HTS/CdS/CdSe/ZnS QDSSCs has been improved. However, it is still less than the values reported by Li et al. [21] (with *η* of 3.86% for the CuS/electrospun carbon nanofiber CE, the higher efficiency due to the good catalytic activity of the CuS nanoparticles as well as the good charge transport provided by the 3D nanofiber framework). We believe that there is still hope for improving the efficiency of QDSSCs by optimizing both the QD deposition time, HTS structure, and developing suitable CuS-based CEs. Larger branched nanorods will lead to a significant increase in the surface areas of the HTS, and this will lead to higher QD adsorptions which in turn lead to an improved solar-to-electric conversion including the superior electrocatalytic activity of CuS by controlled composite films.

To study the electrochemical characteristics of different CEs, we have measured the catalytic activity in the QDSSCs using electrochemical impedance spectroscopy (EIS) (Fig. 7a–e). We find that the impedance spectra of the QD-loaded HTS matched to either a CuS, Cu_2_ZnSn(S,Se)_4_ counter electrode, or Pt counter electrode under forward bias (−0.5 V) and dark conditions are quite different. The Nyquist plot (plot of the imaginary part (Z^''^) vs. the real part (Z^'^) of the impedance) of the EIS of HTS/QDs/electrolyte/CEs (figure inset) appears to be two semi circles which correspond to the charge transfer resistance in high-frequency regions and Nernst diffusion resistance of ${\mathrm{S}}_x^{2-}/{\mathrm{S}}^{2-}$ ions within the electrolyte in low-frequency regions, respectively [12].

From the EIS data, it is clear that the charge transfer resistance (*R*_ct_) decreased in the order: Pt > copper sulfides CEs. The values of *R*_ct_ having and standard Pt, CuS, and Cu_2_ZnSn(S_1 **−** *x*_Se_*x*_)_4_ (*x* = 0, 0.5, and 1) CEs are 2.82 × 10^5^, 80.78, 3.32 × 10^4^, 112.79, and 7.38 × 10^4^ Ω, respectively. The relative lower charge transfer resistance in case of copper sulfides especially on CuS CE (Fig. 7b) reflects superior charge transfer and catalytic ability at the electrolyte/CE interface. Furthermore, the porous structure from the agglomerated nanoparticles of the CuS as seen by SEM image (Fig. 5a) could improve the electrolyte/CuS CE interfacial contact area and increase the possibility for electron transfer compared to those of CEs. However, this tendency breaks for the HTS/QDs/Pt solar cells under the same conditions (Fig. 7a) which exhibited the signature of the capacitive nature of the system. The capacitive nature is due to the buildup chemical potential which is caused by charge accumulation in the surface traps [25]. Based on the performance of the QDSSCs using the CuS counter electrode on the performance of CdS/CdSe/ZnS QD-sensitized HTS photoanode in our study, we obtained only 3.46% of power conversion efficiency with CuS CE, which is near compared to the result from other groups (*η* of 3%) [19–21]. We believe there is hope for more improvement in the efficiency through the optimization of HTS/QDs photoanode by controlling the QD deposition condition. This manifest is responsible for the performance of ayered QD-sensitized solar cells. In addition, the improving catalytic activity for redox couple by controlling thickness, morphology, and conductivity of CuS-based counter electrode such as a composite of copper sulfide/carbon and hybrid metal sulfides are the main key issue investigation. These parameters influence to increase in the electrocatalytic activity and noticeable improvement in the power conversion efficiency QDSSCs [19, 21].

## Conclusions

In present study, the synthesis of hierarchical TiO_2_ spheres (HTS), the use of HTS photoanodes co-sensitized with CdS/CdSe/ZnS quantum dots, and the replacement of the standard Pt CE by CuS and Cu_2_ZnSn(S_1 **−** *x*,_Se_*x*_)_4_ (*x* = 0, 0.5, 1.0) CEs are investigated. After using the CdS/CdSe/ZnS QDs on the HTS photoanodes, it is seen that the light absorption shift to higher wave lengths and a noticeable improvement in the power conversion efficiency occurred when CuS and Cu_2_ZnSn(S,Se)_4_ instead of Pt is used. The actual improvement is an increase of *η* = 3.46% for the CdS/CdSe/ZnS-co-sensitized HTS electrode employing a CuS CE. The ratios of S/Se are found to play a crucial role in determining the power conversion efficiency and electrocatalytic activities for reduction of ${\mathrm{S}}_x^{2-}$ electrolyte. Maximum efficiency of 2.64% is obtained for CZTS_0.5_Se_0.5_ (*x* = 0.5) CE. In our study, the CdS/CdSe/ZnS-co-sensitized HTS electrode employing CuS and Cu_2_ZnSn(S,Se)_4_ CEs show higher energy conversion efficiency than that obtained using Pt as the CE (*η* = 0.37%). To explain this, we propose that the CdS/CdSe/ZnS QD-sensitized HTS coupled to a CuS or Cu_2_ZnSn(S,Se)_4_ CEs has the higher electrocatalytic activity compare to that when Pt CE is used.
